# Melatonin is a potential inhibitor of ovarian cancer: molecular aspects

**DOI:** 10.1186/s13048-019-0502-8

**Published:** 2019-03-26

**Authors:** Hadis Zare, Rana Shafabakhsh, Russel J. Reiter, Zatollah Asemi

**Affiliations:** 1 Research Center for Biochemistry and Nutrition in Metabolic Diseases, Kashan University of Medical Sciences, Kashan, I.R Iran; 2 Department of Cellular and Structural Biology, University of Texas Health Science, Center, San Antonio, TX USA

**Keywords:** Melatonin, Ovarian cancer, Signaling pathways, Anti-inflammatory activities, Anti-angiogenetic properties

## Abstract

Ovarian cancer is one of the most common causes of morbidity related to gynecologic malignancies. Possible risk factors are including hereditary ovarian cancer, obesity, diabetes mellitus, alcohol consumption, aging, and smoking. Various molecular signaling pathways including inflammation, oxidative stress, apoptosis and angiogenesis are involved in this progression of ovarian cancer. Standard treatments for recently diagnosed patients are Surgery and chemotherapy such as co-treatment with other drugs such that the exploitation of neoadjuvant chemotherapy is expanding. Melatonin (N-acetyl-5-methoxy-tryptamine), an endogenous agent secreted from the pineal gland, has anti-carcinogenic features, such as regulation of estradiol production, cell cycle modulation, stimulation of apoptosis as well as anti-angiogenetic properties, anti-inflammatory activities, significant antioxidant effects and modulation of various immune system cells and cytokines. Multiple studies have shown the significant beneficial roles of melatonin in various types of cancers including ovarian cancer. This paper aims to shed light on the roles of melatonin in ovarian cancer treatment from the standpoint of the molecular aspects.

## Introduction

Ovarian cancer is one of the most common causes of morbidity related to gynecologic malignancies [1]. This illness is frequently diagnosed in advanced stages [2] and can be divided into at least five histological types that are characterized by recognizable risk factors, molecular manifestations and clinical properties [3]. Ovarian tumors are graded into 3 types including benign, borderline malignant or malignant [4]. Possible risk factors are including hereditary [5], obesity [6], diabetes mellitus [7], alcohol consumption [8], aging [9], and smoking [10]. Due to absence of noticeable early symptoms in this cancer such as lack of special pelvic or gastrointestinal symptoms [11] its morbidity is high [12]. Standard treatments for recently diagnosed patients are surgery and chemotherapy such as co-treatment with carboplatin and paclitaxel. Although the exploitation of neoadjuvant chemotherapy is expanding [13], treatment in high-grade serous ovarian carcinoma remains a clinical challenge [14].

Melatonin (N-acetyl-5-methoxy-tryptamine), an endogenous agent secreted from the pineal gland [15], transfers information about period of darkness to all cells [16] which its peak is at highest level among 2AM and 5AM [17]. Melatonin has a critical role in determining homeostasis, neurohumoral stableness and circadian rhythms through synergetic activities with other hormones and neuropeptides [18]. Melatonin is classified as an autocoid, a chronobiotic, a sleep-inducing agent, an immune modulator and a biological adjusting agent. In addition, melatonin has anti-carcinogenic features, such as regulation of estradiol production, cell cycle modulation, upgrading apoptosis [19] as well as anti-angiogenetic properties [20], significant antioxidant effects [21] and modulation of various immune system cells and cytokines [22]. There is considerable evidence of its ability in prevention and treatment of cancers [23]. Multiple studies have shown significant beneficial roles of melatonin in various cancers types such as breast cancer [24] pancreatic cancer [25] lung cancer [26] and ovarian cancer [27]. A retrospective study showed that melanin levels are lower in women with ovarian cancer compared with healthy ones (41.8 versus 82.4 pg/mL) [28]. Even though there is no significant relationship between urinary melatonin levels and ovarian cancer risk in women, several evidence shows that melatonin have potential therapeutic properties against this cancer. A recent in vivo study reported that melatonin treatment for 60 days potentially decreased ovarian cancer mass without any peritoneal adhesions and tumor incidence including high-grade serous papillary, sarcoma and undifferentiated carcinoma [29]. In addition, an in vitro study observed that response to melatonin in various types of ovarian cancer cells was different. In fact, there was a non-heterogeneous response to melatonin as one of the cell lines inhibited by 90% while another one inhibited by 30% [30]. This report sheds the new light on the roles of melatonin in prevention, treatment and chemotherapetic interplay in ovarian carcinoma as identified in recent studies and clinical trials.

## Ovarian cancer pathogenesis

Although some causes of ovarian cancer remain unclear, several hypothesizes have been advanced. One is the incessant ovulation hypothesis which proposes the formation and progression of ovarian cancer throughout cyclical ovulatory processes. This hypothesis suggests that replicative DNA mistakes are increased in ovarian epithelial cells during follicular growth and ovulation [31]. Another hypothesis is the gonadotrophin theory suggesting that gonadotrophins lead to excessive proliferation of ovarian epithelial cells which result in tumor formation [32]. Hormonal influences are the third hypothesis which suggests the effects of hormones such as androgen and progesterone on proliferation of the ovarian epithelial cells and therefore ovarian cancer formation [33]. The surface of the ovarian epithelium is part of the peritoneal lining and, therefore, is exposed to substances which exist in the peritoneal cavity. Most of these substances have inflammatory features. A primary physiological role of the ovary is ovulation, which has pro-inflammatory properties [34]. During the ovulatory processes followed by immediate ovum release, a large number of molecules are generated including cytokines and chemokines, prostaglandins, plasminogen activators, bioactive eicosanoids, interleukins, collagenases, tumor necrosis factors, several growth factors and also various immune cells which all activate a pro-inflammatory cascade. Some of these pro-inflammatory molecules including CCL2/MCP-1, CCL5/RANTES and IL-8 are activated during cyclical ovulation; thus, the incessant ovulation theory suggests that inflammation along with other physiological conditions enhances the progression of ovarian cancer [35]. Conversely, as suggested by the gonadotrophin and hormonal hypothesizes, increased estrogens and androgens recruit several pro-inflammatory cells and molecular stimulators leading to immune activation [36]. Collectively, these hypothesizes indicate the influences of ovulation, gonadotrophin and hormonal changes on formation and progression of ovarian cancer which are all related to activation of inflammatory mediators as well as persistent creation of genomic damages.

## Melatonin and ovarian cancer; molecular mechanisms

Treatment by melatonin leads to a reduction in various proteins involved in ovarian cancer signaling pathways including oxidative stress, inflammation, apoptosis, cell cycle and proliferation. One of these molecules is E-cadherin which is a tumor suppressor and a key molecule for sustaining adherent junctions in cell surface [37]. Up-regulation of E-cadherin expression in ovarian cancer tissues has prognostic value to distinguish tumors in late and early stages [38]. Melatonin increases E-cadherin in ovarian cancer cells [39]. Another factor modulated by melatonin is the estrogen receptor α (ERα). This molecule is one a member of nuclear receptor super family and modulates cell multiplication, homeostasis, and differentiation in multiple tissues. Sustained exposure to estrogen/estradiol (E2) up-regulates the growth of ovarian cancers [40]. Melatonin is a special ERα suppressor [41] and plays an anti-carcinogenic role through the estrogen receptor (ER) pathway in tumor cells [42].

## Antioxidant effects of melatonin

There is agreement that melatonin is an important endogenous free-radical scavenger [43] and possesses several antioxidant roles by influencing the electron transfer chain, preventing peroxynitrite levels via regulation of nitric oxide synthases (iNOS, nNOS) and thereby reducing NO levels. Melatonin enhances the intra-mitochondrial anti-oxidative potential by improving glutathione levels and promoting glutathione peroxidase, manganese-superoxide dismutase (Mn-SOD) in the matrix and copper, zinc (Cu, Zn-SOD) in the inter-membrane space [44]. Melatonin treatment leads to ROS reduction and activation of several anti-oxidant enzymes such as superoxide dismutase, catalase and glutathione [45]; melatonin also acts as a pro-oxidant in different cancers [46]. In pre-ovulatory follicular fluid, melatonin alleviates the carcinogenic effect of ROS in follicular fluid [47]. Some studies revealed that cyclooxygenase-2 (COX2) is over-expressed in tumor cells. Melatonin suppresses COX2 activity [48] leading to prevention of DNA damage [49].

## Melatonin and apoptosis

Apoptosis, a type of programmed cell death, has both specific morphological features and biochemical mechanisms. Melatonin regulates apoptosis in several types of cancers by multiple mechanisms. Caspases are interleukin-1beta-converting enzyme family members which are also aspartate-specific cysteine proteases [50] and have pivotal roles in regulation of the initiation, transduction and promotion of apoptotic signals. In ovarian cancer the expression of cleaved caspase-3 is increased [51] while melatonin reduces the over-expression and activation of this molecule [52]. Thus melatonin administration enhances apoptosis in ovarian cancer cells [53].

Evidences suggest that treatment with melatonin enhances apoptosis by increasing the expression of p53, a tumor suppressor, in ovarian cancer cells [53]. P53 signaling pathway in various carcinoma cell lines acts significantly as a transcription factor, resulting in arrest cell cycle or apoptosis [54] by inhibition of the cell cycle in the G2-phase [55]. Accumulated p53 significantly interacts with proteins which are important in tumor cell maintenance and thereby neutralizes them [56]. Melatonin increases [57] and activates p53 and therefore increases apoptosis in several cancers such as the colon [58] and the uterus [59]. Two important members of apoptosis-related genes are Bcl-2 and BAX [60]. Melatonin motivates BAX gene expression, and down regulates the expression of anti-apoptotic gene BCL-2 [61, 62]; thus, melatonin regulates the Bax/Bcl-2 ratio [63]. A recent study suggested the role of melatonin in induction of apoptosis in ovarian cancer cells by increasing BAX expression and reducing in Bcl-2 levels [53].

## Melatonin as an anti-inflammatory agent

In addition to its anti-oxidant effects melatonin’s anti-inflammatory and immune regulatory properties are well documented. Melatonin induces the release of interleukin-2, interleukin-10 and interferon-γ which leads to the enhancement of T-helper cells which respond to these substances. T-helper cells have a significant anti-cancer role. Nuclear factor-kappa B (NF-kappa B) enhances ROS generation leading to DNA damage [64, 65]. In the etiology of the ovarian cancer, NF-kB is a significant marker of inflammation [66]. Melatonin suppresses the NF-kB phospho-activation [67]. Furthermore, melatonin decreases H_2_O_2_-induced oxidative stress by regulation of Erk/Akt/NFkB pathway [68]. Melatonin therapy down-regulates the mRNA expression of NFκB1, NFκB2 in mice [69]. This indolamine also inhibits the expression of TNF-α which is an important member of TNF/TNFR cytokine super-family; via these mechanisms melatonin acts as an anti-inflammatory agent [70]. TNF-α is a pro-inflammatory agent which causes pathological processes such as chronic inflammation and malignancy [71]. In ovarian cancer cells the expression of TNF-α is elevated [72]. Melatonin administration importantly inhibits this increase in ovarian cancer cells. HER-2 is another factor involved in the initiation and maintenance of inflammation and tumorogenesis in cancer cells. HER2 initiates a feed-forward activation circle of IL-1α and IL-6 that induces NF-κB and STAT3 pathways for induction and preservation of cancer cells [73]. Her2 stimulates kinases and transcript agents which support cancer medicine resistance and metastasis. Melatonin critically suppresses this invasive/metastatic aspect; the mechanism involves the repression of mesenchymal-to-epithelial transition, either by helping mesenchymal-to-epithelial conversion, and/or by obstructing important signaling pathways implicated in later stages of metastasis [74]. Melatonin also regulates Her-2 system in invasive tumors by decreasing the Her-2 expression [75]. Signaling pathways of transforming growth factor-β (TGF-β) have significant roles in ovarian cancer [76]. TGF-β may enhance cell survival by positive modulation of the cell cycle as well as by preventing apoptosis [77]. The expression of TGF-ß1 and its receptors may play an important role in promotion and proliferation of tumor cells [78]. Melatonin prevents [79] and decreases the expression of TGF-β1 in epithelial ovarian cancer [80]. Collectively, the findings indicate that melatonin administration significantly regulates signaling pathways in ovarian cancer [81].

## Melatonin and its ani-angigenesis effects

One of important aspects of metastatic spread and proliferation of cancer cells is presence of necessary nutrients and oxygen and also should be removed waste products [82, 83]. In this regards, vascular network and new growth of vessels are known as well players for these actions. New lymphatic and blood and vessels form via processes which are known as lymphangiogenesis and angiogenesis respectively [84]. Multiple lines evidence revealed that angiogenesis could be modulated by both inhibitor and activator molecules. Several proteins have been observed as angiogenic inhibitors and activators. Despite many efforts, antiangiogeic inhibitors have not documented useful in terms of long-term survival. Therefore, there is a serious require for finding, and developing new effect therapeutic platforms combining anti-angiogenic therapies along with conventional cytoreductive treatments in the management of different cancers [83]. Vascular endothelial growth factor (VEGF) is significantly over-expresses in cancer patients [85, 86] VEGF inhibits apoptosis, protects tumor and vascular growth, and enhances proliferation and inflammation leading to carcinogenesis [87]. Melatonin decreases VEGF secretion resulting in inhibition of angiogenesis in tumors [88]. Melatonin also was shown to inhibit angiogenesis by decreasing angiopoietins and VEGF in an animal model of ovarian cancer [89].

Besides different factors which are associated to over expression and activation of pro-angiogenic growth factors and their receptors, hypoxia has been emerged as key factor. Given that cells employed a variety of genes to adapt to hypoxia in low-oxygen positions. Among of these genes, HIF-1 is major and primary players in hypoxic conditions [90, 91]. HIF-1α and HIF-1β are well-known subunits of HIF-1. These subunits are known as a basic helix-loop-helix (bHLH) transcription factor family. It has been showed that HIF-1α/HIF-1β dimer has various targets such as VEGF. Hence, this protein is related to increasing of angiogenesis. Along to different signaling pathways involved in angiogenesis, STAT3 is another angiogenesis factor which is able to elevate the expression of VEGF and stimulate HIF-1α stability. Increasing evidence indicated, when STAT3 and HIF-1α link to CBP/p300 (is known as a co-activator in the VEGF promoter); there were normal activation of VEGF transcription [88]. The activated STAT3 is associated with initiation and progression of several malignancies such as ovarian cancer, and melanoma. It has a crucial role in different biological processes including survival, proliferation of cells, migration, invasion, and angiogenesis [92]. As mentioned above, melatonin shows anti-cancer activities such as anti-angiogenesis property. In this regards, given that melatonin enables to effect on angiogenesis thought targeting HIF-1α under hypoxic conditions [46, 93]. In a study, Park et al. [94] revealed that there is a decrease for HIF-1α and pVHL binding during hypoxic conditions in colon cancer cell lines. While the presence of melatonin is able to recover PHD activity in the treatment group and then elevated the binding of HIF-1α and pVHL. In another study, Zhang et al. [95] documented melatonin increases binding of pVHL and HIF-1α during hypoxia in glioblastoma cells. Taken together, expression levels of angiogenic factors are associated with the tumor cells aggressiveness. Thus, identification of new angiogenic inhibitors (e.g., melatonin) could contribute to decrease both mortality and morbidity from carcinomas.

## Melatonin and metabolic alterations in ovarian cancer

Alteration in cancer cell metabolism is one of the important events occurred during tumorogenesis and cancer progression. Cancer cells need to be able to proliferate and growth in a hypoxic and nutrient-poor microenvironment which require a reprogramming of metabolism in these cells, especially the key metabolic substrates such as glucose, lipid and etc. in this way, mitochondria gets more functional changes among other organelles. Despite of the high glycolytic rate in cancer cells, the produced pyruvate is not used in Krebs cycle and it is transformed into lactate independently from oxygen availability in the so-called Warburg effect [96]. In addition, many clinical studies reported that plasma glucose levels in cancer patients is highly elevated and may be a significant prognostic indicator for cancer. Besides, some recent studies demonstrated that the expression of glucose transporter protein 1 (GLUT1) increased in ovarian cancer cells leading to elevation of glucose uptake in these cells [97]. Recently, new beneficial therapeutic targets which involve in cellular pathways responsible for energy generation required to control cancer cell growth are emerging. Several studies showed that melatonin, at both physiological and pharmacological concentrations, is able to regulate cellular metabolism through different mechanisms [98]. It has been proved that melatonin crosses cell membranes via glucose transporters which may lead to reduction of glucose uptake in cancer cells [99]. In addition, melatonin is able to decrease the production of lactate, but the certain mechanism which in how melatonin affects glycolysis is not clear yet [100]. Moreover, melatonin influences insulin secretion from pancreas by its MT1 and MT2 receptors leading to reduction in blood glucose and elevation in fatty acids [101]. Recent findings showed that melatonin reduced proteins related to metabolic systems including production of several metabolites and energy, endoplasmic reticulum stress related pathways, cancer-associated proteoglycan, HIF-1 signaling and antigen processing in ovarian cancer. Indeed, melatonin down-regulates several proteins related to metabolism including glyceraldehydes-3- phosphate dehydrogenase, pyruvate kinase isozymes M1/M2, fructose-bisphosphate, aldolase A, lactate dehydrogenase A chain, creatine kinase B, protein disulfide isomerase A3 and A6, subunit *α* of ATP synthase, 78-kDa glucose-regulated protein and peptidyl-prolyl cis-trans isomerase A. these alterations in metabolism may significantly influence aerobic glycolysis leading to reduction in proliferation and metastasis in ovarian cancer cells. Besides, melatonin overexpressed some molecules including subunit β of ATP synthase, fatty acid binding protein and 10-kDa heat chock protein in ovarian cancer cells [81].

## Melatonin as an adjuvant therapy in ovarian cancer treatment

Recent investigations have described the role of melatonin in combination with radio- or chemotherapy in several cancers including ovarian cancer [102]. These studies also showed that the optimal dose of melatonin is safe and effective in enhancing radiotherapy ratio therapeutic effects as well as providing radioprotection [103]. Melatonin up regulates the tolerance of normal tissues to toxic effects of ionizing radiation in patients who undergo radiotherapy by enhancing DNA damage responses and reducing the risk of instability. Melatonin plays synergistic roles in radiotherapy and chemotherapy as an antioxidant which relieving the side effects of these destructive treatments [104].

In ovarian cancer this powerful antioxidant potentially supports the ovaries against damage induced by cisplatin; this is the main chemotherapeutic agent for this cancer [105]. Recently it has been shown that melatonin improved fertility in ovarian cancer [106] via the promotion of ERK/p90RSK/HSP27 cascade in SK-OV-3 cells. Moreover, melatonin and cisplatin co-administration promoted apoptosis induced by cisplatin. It was also demonstrated that melatonin improved cis-diamminedichloroplatinum sensitivity in HTOA and OVCAR-3 cells; both these lines are ovarian cancer cells [107]. Other investigations also showed that melatonin co-administration potentially boosted laser effectiveness by induction of apoptosis in ovarian tumor cells leading to significant improvement of apoptosis/necrosis ratio, and also increasing the expression of heat shock protein 70 compared to administration each factor alone [108]. Thus, melatonin acts as a powerful synergistic agent with cisplatin therapy [109] and can be applied as an adjuvant in ovarian cancer treatment.

## Conclusions

**Fig. 1:**
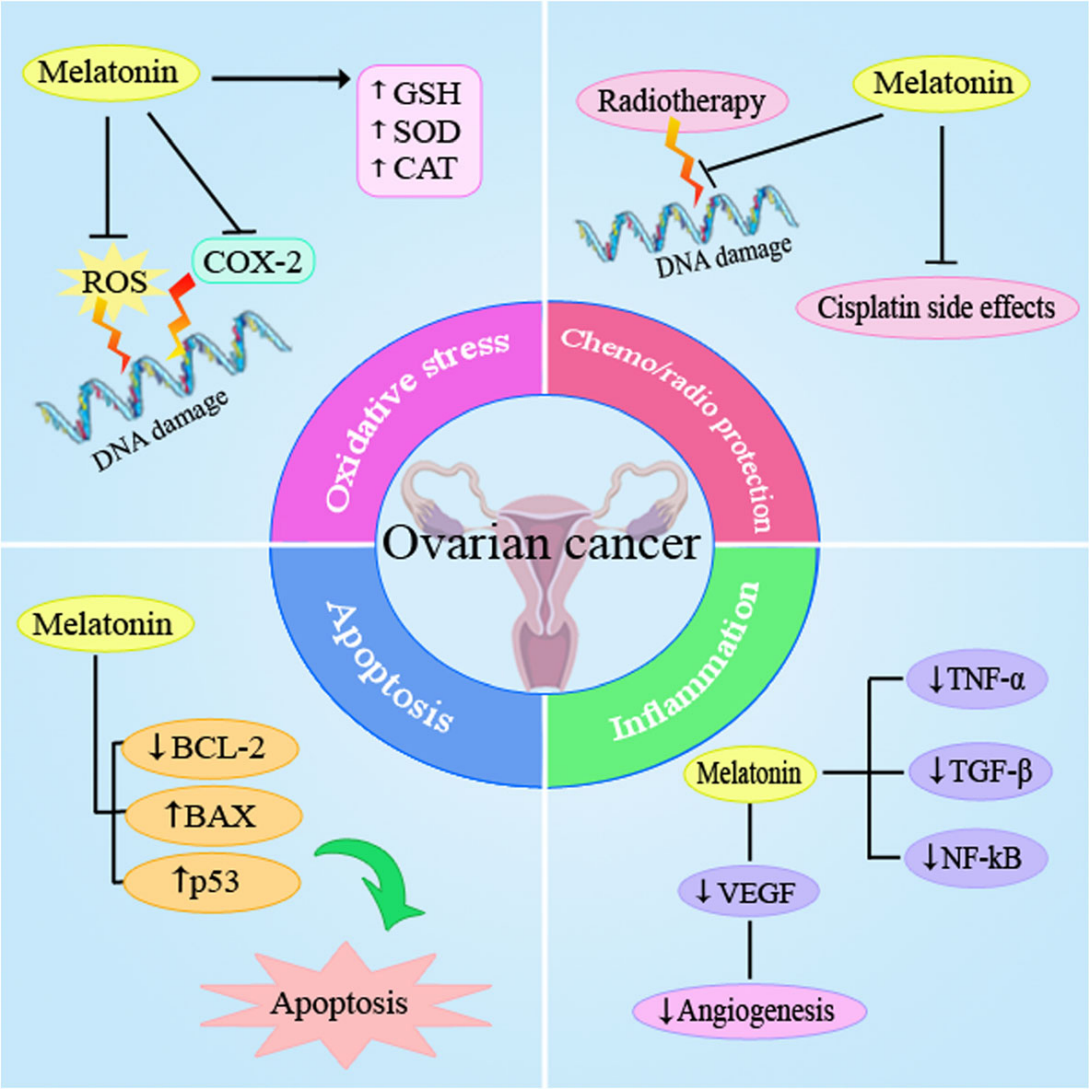
Schematic representation in targeting different signaling pathways using melatonin as a novel therapeutic strategy in the treatment of ovarian cancer

Ovarian cancer is a gynecologic malignancy with a high morbidity rate. The pathogenesis of this cancer is complex from its molecular aspects. Several factors and various molecular signaling pathways such as inflammation, oxidative stress, apoptosis and angiogenesis are involved in its progression. There is a large amount of evidence that documents the potential beneficial effects of melatonin in inhibition of development and progression of ovarian cancer via its multiple potential features including antioxidant, anti-inflammatory, metabolic effects and apoptosis induction activities in these tumor cells **(**Fig. 1). Also melatonin has beneficial effects with several anti-cancer drugs such as cisplatin. Thus, it is suggested that melatonin can be co-administrated as a potent adjuvant agent in combination with other chemotherapeutic drugs in ovarian cancer treatment. Current therapeutic strategies for ovarian cancer are often limited and evidence has shown that chemotherapy alone is not completely efficient to reduce tumor cells. Thus, finding new therapeutic options with low adverse effects is considerable. Melatonin is endogenously produced and its pharmacological doses are available with no toxicity. Although these potential roles have long been known, it has not been fully exploited in clinical trials. Melatonin as a natural molecule with its potential anti-cancer properties and its efficiency in decreasing side effects of current treatments may be an appropriate option in the treatment of ovarian cancer.

## Abbreviations

ERα: estrogen receptor α
TGF-β: transforming growth factor-β
